# Development and laboratory evaluation of a novel IoT-based electric-driven metering system for high precision garlic planter

**DOI:** 10.1371/journal.pone.0317203

**Published:** 2025-01-17

**Authors:** Abdallah Elshawadfy Elwakeel, Ahmed Elbeltagi, Ahmed Z. Dewidar, Ali Salem, Mohamed Anwer Abdeen

**Affiliations:** 1 Faculty of Agriculture and Natural Resources, Department of Agricultural Engineering, Aswan University, Aswan, Egypt; 2 Faculty of Agriculture, Agricultural Engineering Department, Mansoura University, Mansoura, Egypt; 3 Prince Sultan Institute for Environmental, Water and Desert Research, Prince Sultan Bin Abdulaziz International Prize for Water Chair, King Saud University, Riyadh, Saudi Arabia; 4 Department of Agricultural Engineering, College of Food and Agriculture Sciences, King Saud University, Riyadh, Saudi Arabia; 5 Faculty of Engineering, Civil Engineering Department, Minia University, Minia, Egypt; 6 Faculty of Engineering and Information Technology, Structural Diagnostics and Analysis Research Group, University of Pécs, Pécs, Hungary; 7 College of Engineering, South China Agricultural University, Guangzhou, China; 8 Agricultural Engineering Department, College of Agriculture, Zagazig University, Zagazig, Egypt; G H Raisoni College of Engineering and Management, Pune, INDIA

## Abstract

In order to address many issues, such as the inconsistent and unreliable seeding process in traditional mechanical garlic seed metering systems (SMS), as well as the lack of ability to monitor the effectiveness of the seeding, a highly accurate electric-driven metering system (EDMS) was developed and created specifically for garlic seed planters. This study provided a description of the overall structure and functioning principle, as well as an analysis of the mechanism for smooth transit and delivery. A combination of an infrared (IR) sensor, Arduino Mega board, stepper motor, speed sensor, and a Wi-Fi module was employed to operate the EDMS, as well as monitor and count the quantity of garlic seeds during the planting process and determine the qualified rate (QR) and missing rate (MR). A monitoring system of the planting quality of garlic seeds was created based on the IoT concept. Then, the performance of the EDMS was validated in a laboratory setting utilizing a bench test at six operating velocities of 10, 20, 30, 40, 50, and 60 rpm of the EDMS. The obtained results showed that the correlation coefficient between the actual and detected garlic seed using the garlic seed monitoring and counting system (GSMCS) was 0.9723. Additionally, the EDMS observed a maximum QR of 96.23% at an operating velocity of 20 rpm, with a standard division and standard error of 1.61030 and 0.72015, respectively. Additionally, the EDMS minimized the MR up to 3.77% at the same operating velocity, with standard division and standard error of 1.65325 and 0.73936, respectively. Furthermore, the results indicated a progressive increase in the QR and MQ standard errors as the EDMS’s operating velocity increased. Additionally, the sensor’s monitoring accuracy gradually declined with an increase in the operating speed of the EDMS. Finally, this study introduced a novel EDMS to garlic seed planters that was not used before. The developed EDMS and GSMCS are technical manuals for developing and designing monitoring systems capable of precisely measuring and identifying the rates of qualifying and missing garlic seed measurements.

## 1. Introduction

Currently, the garlic business is undergoing a significant transition from conventional to new methods [1,2], Western European countries and Japan have a high level of mechanization in garlic production [3,4]. The 2030 Agenda for Sustainable Development was endorsed by world leaders in September 2015 during the United Nations summit. In line with the zero-hunger objective outlined in sustainable development, there is advocacy for a rise in agricultural productivity and efficiency. Automating the process of planting garlic can efficiently reduce the need for manual labor and enhance productivity. The current garlic sowing machines exhibit substantial variability in seed rates, including both excessive and insufficient seeding, as well as inadequate accuracy in upright positioning. These issues arise from the impact of garlic seed kinds and their morphological properties [5,6]. Investigating the use of a single SMS for garlic seeding is a crucial aspect of mechanizing garlic sowing. This approach can effectively address the performance issues associated with garlic sowing machines and enhance the mechanization efficiency of garlic planting [7]. Garlic planting in European and American countries necessitates straightforward mechanization, random seeding with wide row spacing, and the use of rather heavy equipment. The majority of those individuals utilize spoon-chain SMS [8,9]. Garlic growing in China necessitates agronomic practices such as narrow spacing between rows, high planting density, and the use of single seeds sown in an upright position. China uses minimal mechanization and scales down the size of its equipment. Spoon-chain, rotary-table, and vibrating seed-measuring machines are extensively utilized [10,11].

Extensive research has been undertaken on enhancing the precision and effectiveness of SMS used in garlic seeding equipment, where an automatic garlic planter was designed and tested by Geng et al. [12]. Where the garlic SMS consists of a chain drive, seed-taking spoon, chain plate, and vibrating mechanism. The experimental results indicated that the QR and MR were 93.50% and 4.20%, respectively; in the study conducted by Guo et al. [6], a garlic SMS was designed, and the performance was simulated at a velocity range of 15–35 rpm. The obtained results showed that the QR was over 80% for a single SMS; also, a self-propelled garlic planter was constructed and assessed by Nare et al. [13]. The obtained results showed that the missing index, multiple indexes, and seed damage were found to be only 2.67, 8.0, and 1.46%, respectively; additionally, a type of hole-wheel garlic SMS was designed and experimented on by Li et al. [14]. The bench test results showed a QR and MR of 89.2 and 5.0%, respectively; furthermore, a chain spoon garlic SMS was proposed and optimized by Li et al. [15]. The field trials revealed that the QR was 89.2%, and there was a significant difference in the MR throughout the operation, requiring hand replanting; finally, in the study conducted by Feng et al. [16], the details of the design and experimental testing of a pneumatic garlic SMS were described. Bench tests showed that the QR and MR were 88.04% and 8.65%, respectively.

At present, precision agriculture technology (PAT) is becoming increasingly popular as a financially viable and ecologically sustainable method. PAT utilizes crop-related data collected from several sensors to accurately apply the necessary inputs, such as seeding machines, in order to minimize both environmental impact and expense [17–20]. Given the advantages of seed conservation, reduced labor intensity, and enhanced operational efficiency, precision seeding and planting will be a crucial focus for agricultural innovation in the forthcoming years [21–23]. PAT is a crucial factor for the planting process at the ideal time, at consistent intervals. Grain seeds must be evenly dispersed around the field and sown at the optimal seeding rate per heactar to achieve the desired crop rows. Furthermore, optimizing plant-to-plant spacing enhances crop output [24–26]. Variable rate application (VRA) has gained traction alongside precision planting in smart agriculture. VRA’s stated goal is to maximize fertilizer, seed, and pesticide savings by adhering to field-specific principles of site-specific management [27]. PAT comprises a series of distinct stages. Firstly, data collection pertaining to the crops and their surrounding environment is conducted. Subsequently, crop conditions are assessed, and viable solutions are derived from the acquired data. The next step involves the recommendation of specific actions for crops based on the identified solutions. Lastly, an analysis of the outcomes resulting from the recommended actions is carried out [28]. The Internet of Things (IoT) is a critical concept within the realm of PTA and information technology. It represents a cutting-edge technological advancement that involves the transformation of physical objects into intelligent virtual entities, thereby exerting a significant influence on the trajectory of technological advancement. The core objective of this concept is the seamless integration of all components of our environment into a unified infrastructure. This integration not only enables the control of our surroundings but also facilitates real-time access to pertinent information regarding its state [29]. Researchers around the world have extensively employed IoT technology in various agricultural domains, including the study of soil nutrients [30–33], drying of agriproducts [34–39], environmental control in poultry houses [40], smart farm machinery [41–51], fruit sorting [52], and intelligent and smart irrigation systems [53–56].

Historical research on garlic planting has predominantly relied on mechanical means to transmit motion from the ground wheel to the seeding mechanism. However, this method does not fulfill the precision and intelligent agriculture requirements essential in today’s context. Therefore, the main aim of this study was to develop and validate the functionality of an Internet of Things (IoT)-enabled Electric-Driven Metering System (EDMS) integrated with a Garlic Seeds Monitoring and Counting System (GSMCS). This system was specifically crafted to harness the capabilities of IoT to address the limitations present in the current garlic planting apparatus. The primary goal of this investigation is to contribute to the development of efficient and effective garlic cultivation methodologies. The secondary goal is to reduce the number of garlic seeds required for planting, increase crop yield, and improve the overall quality of the harvested garlic.

## 2. Materials and methods

### 2.1. System components

**Fig 1:**
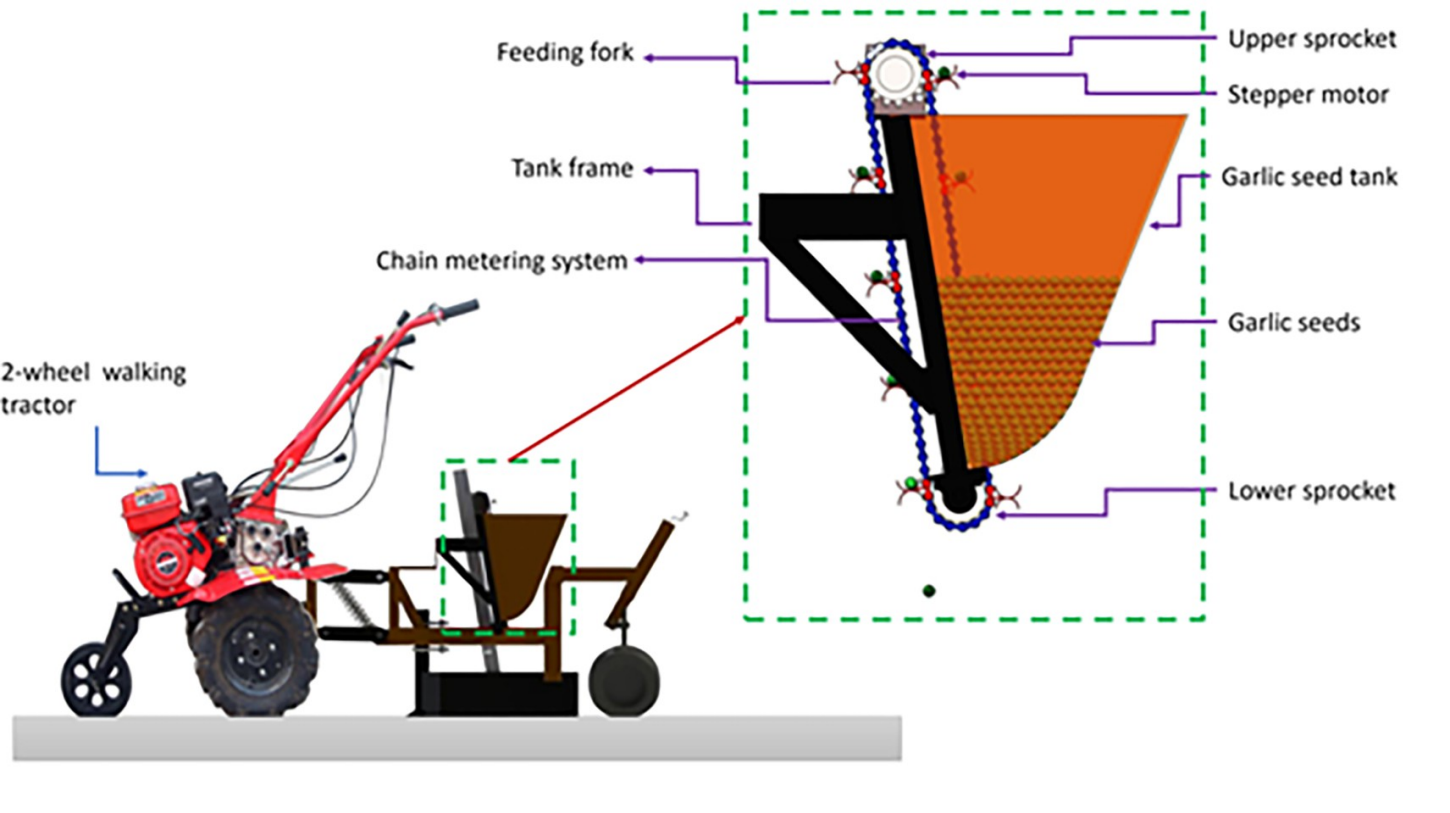
Main components of the planting unit of the garlic seeds.

The main components of the developed EDMS are presented in **Fig 1**. Where the developed EDMS consists of two main parts: 1. Hardware components: including machine frame, mechanical components, and electronic components; 2. Software components: consist of an operating and control system of the EDMS and operating of the GSMCS.

### 2.2. The overall structure and working principle

**Fig 2:**
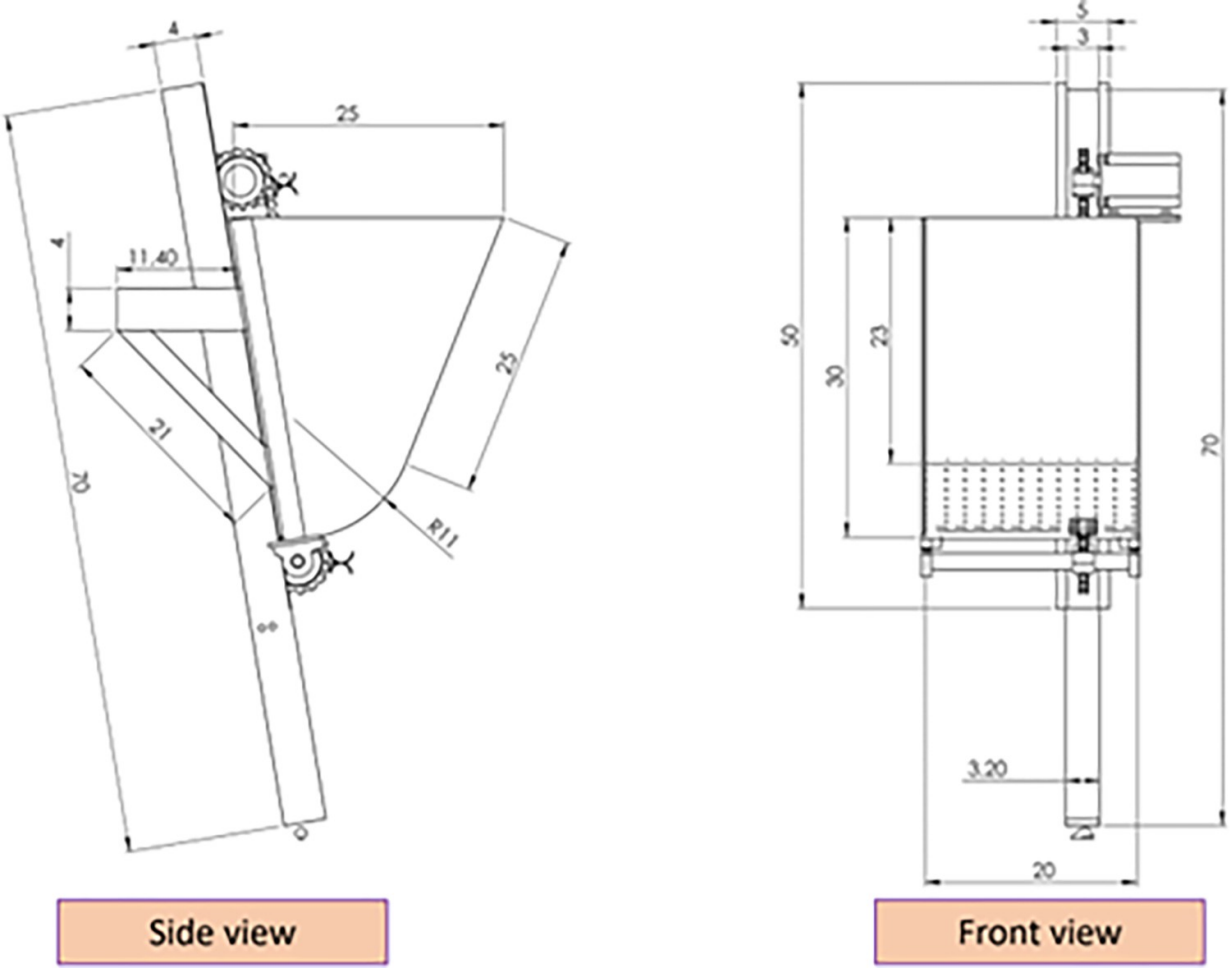
Main dimensions of the planting unit of the garlic seeds (all dimensions in cm).

The present study utilized a developed EDMS for a garlic seed planting unit, illustrated in **Figs 1 and 2**, consisting of a garlic seed tank made of a galvanized metal sheet with a thickness of 1 mm. The seed tank’s primary dimensions, as depicted in **Fig 2**, are 30 cm in height, 20 cm in length, and 25 cm in width. To facilitate the flow of garlic seeds into the EDMS, the tank’s conical shape terminates in a circular bottom. The EDMS was designed using a 1/4 (6.35 mm)-sized, 20 mm-wide chain commonly found in mid-range motorcycles. Feeding forks or feeding cubs with a diameter of 20 mm and a depth of 5 mm were welded onto the lifting chain at intervals of approximately 9 cm. Oppositely oriented feeding forks were welded in pairs, with the upper one elevating garlic seeds from the seed tank to the upper point (at the stepper motor). Following the turning point, the feeding forks rotate, and the garlic seeds are dropped due to gravity, landing in the lower feeding fork to increase planting uniformity while reducing seed damage. The feeding or lifting chain was installed at two sprockets of the same diameter of approximately 10 cm. The upper sprocket was fixed with a stepper motor, while the other sprocket was fixed with a 20 mm pivot shaft suspended with two ball bearings at each end. To minimize losses of garlic seeds and direct the seed to the GSMCS, the chain was covered with a 50 * 30 mm galvanized metal sheet cover.

### 2.3. Design of electronic circuits for EDMS and monitoring system

**Fig 3:**
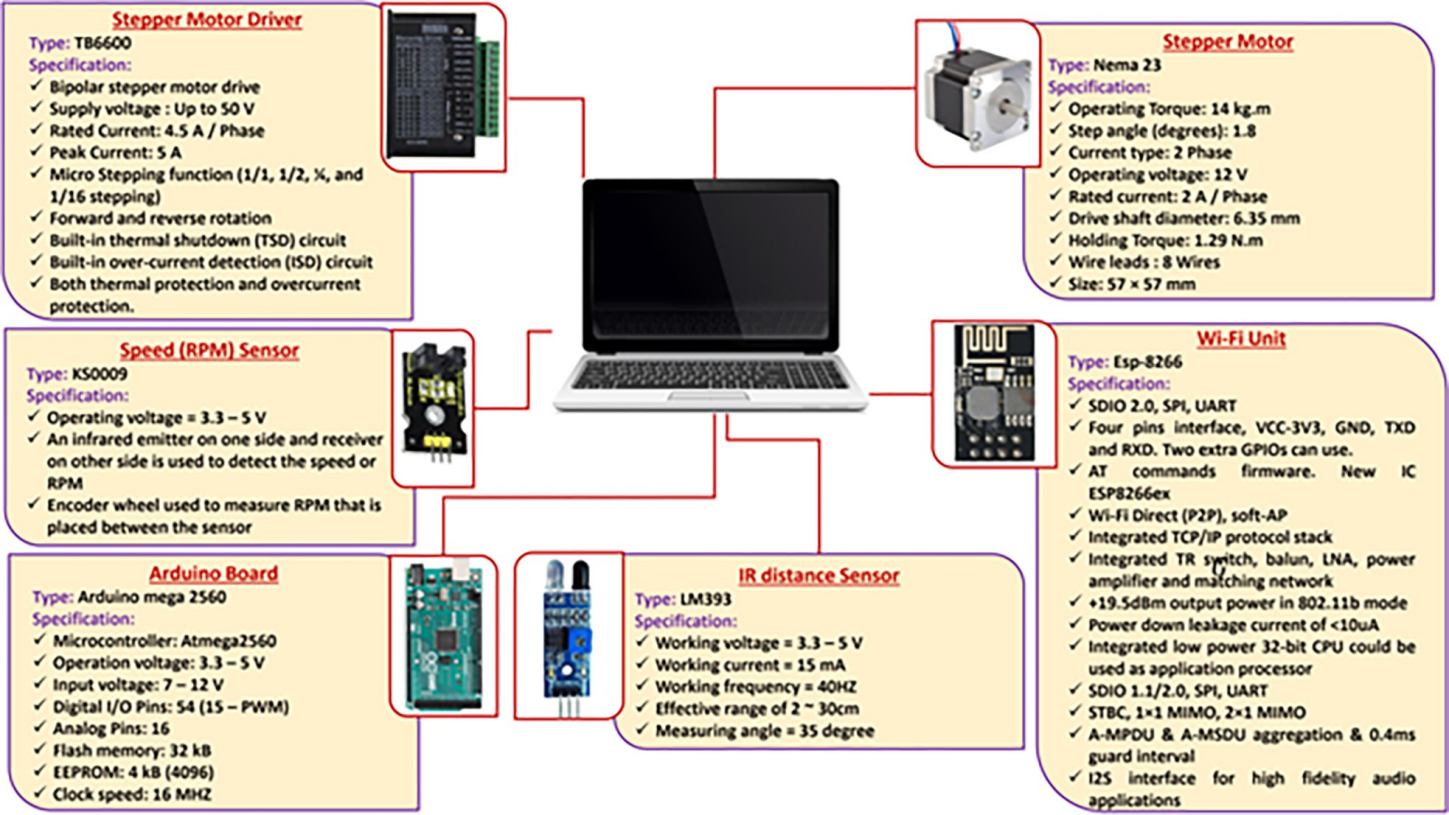
Technical specification of the different software components used for designing the developed EDMS and GSMCS.

**Fig 4:**
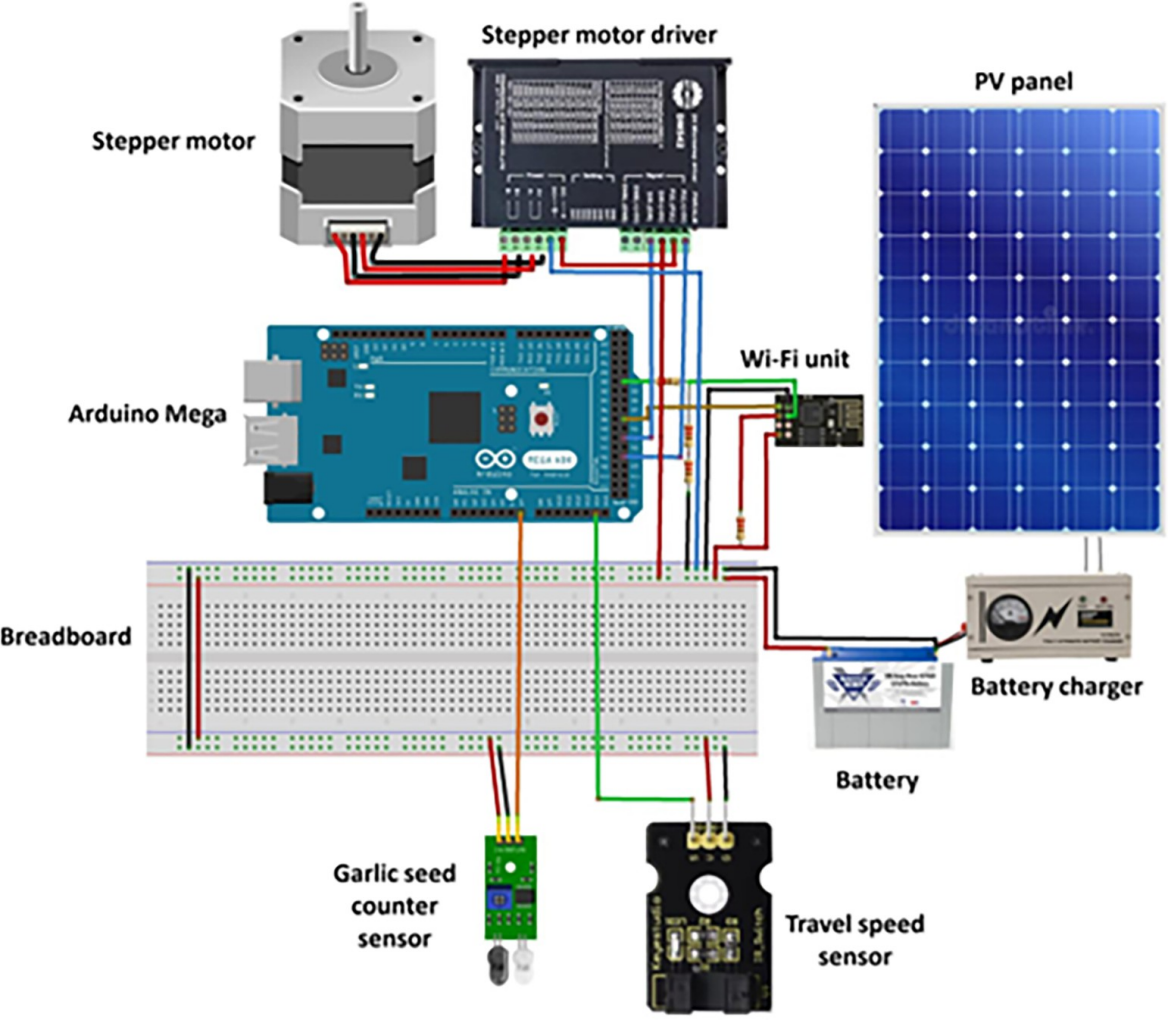
Connection of the electronic circuits for developed EDMS and GSMCS with Arduino mega board.

Both EDMS and GSMCS are sophisticated systems that encompass various electronic components. **Fig 3** illustrates the different elements that have been integrated into the EDMS, and **Fig 4** illustrates the electrical connections of the control and measuring electronic circuits of the developed EDMS. Where the developed system is powered by a photovoltaic system, which contains a PV panel (60 W, India), a battery (70 A.h.), a battery charger (12 V, Netherlands), and a voltage converter (12–5 V, China).

#### 2.3.1. Design of electronic circuit for the developed EDMS

**Fig 5:**
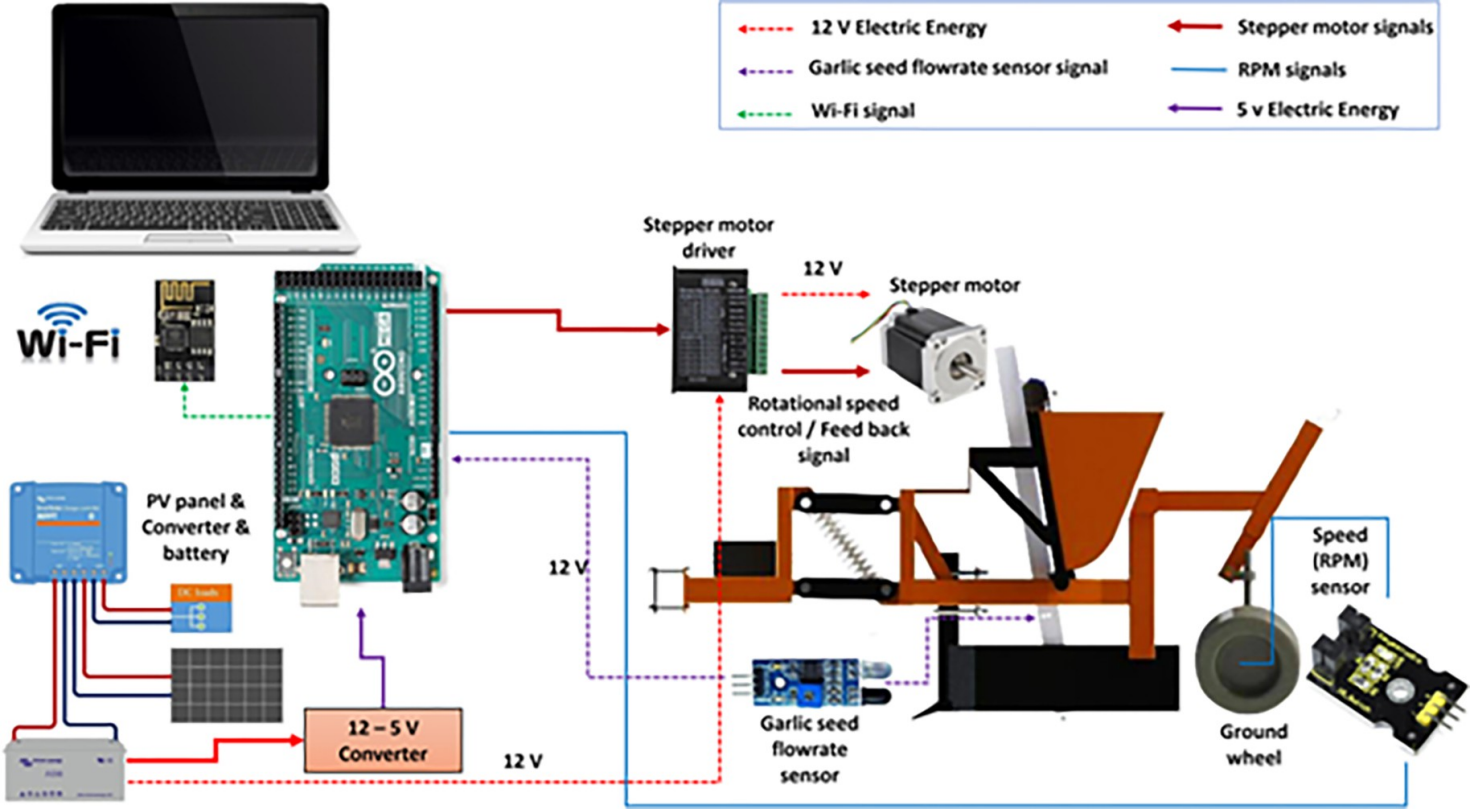
Operating map of the developed EDMS and monitoring system.

As shown in **Fig 5**, the speed sensor (model: KS0009, China) is responsible for detecting the travel speed of the garlic planting system. The EDMS, responsible for lifting garlic seeds, is powered by a stepper motor (model: Nema 23, CUI Devices, Tualatin, USA) with an operating torque of 14 kg.m and a 1.8-step angle. To drive the stepper motor at the desired speed based on the travel speed and planting spacing, a stepper motor driver (model: TB6600, Sorotec GmbH, Germany) with a rate pulse of up to 50 V and a rate current of 4.5 A/pulse was used. The EDMS is equipped with an Arduino Mega board with a processor (model: ATM2560, China) as a microcontroller to obtain signals from the speed sensor. The microcontroller makes decisions based on operating algorithms and sends data to a laptop using the Wi-Fi module. **Fig 5** shows the operating map of the developed EDMS and monitoring system, where the travel speed measured by the speed sensor is integrated with the ground wheel.

During the designing of the variable rate EDMS, we used a speed sensor (model: KS0009, China). Because of this, the sensor be used in various applications where you need to track motion or speed, such as robotics projects involving motion control, controlling conveyor belts, and building tripwire alarms. Where this sensor is designed to measure speed or act as an infrared light break switch. In simpler terms, it detects objects passing through a slot by interrupting an infrared beam and can be used to measure the travel speed of the developed machine.

#### 2.3.2. Analysis of the mechanism for stable transport and delivery

**Fig 6:**
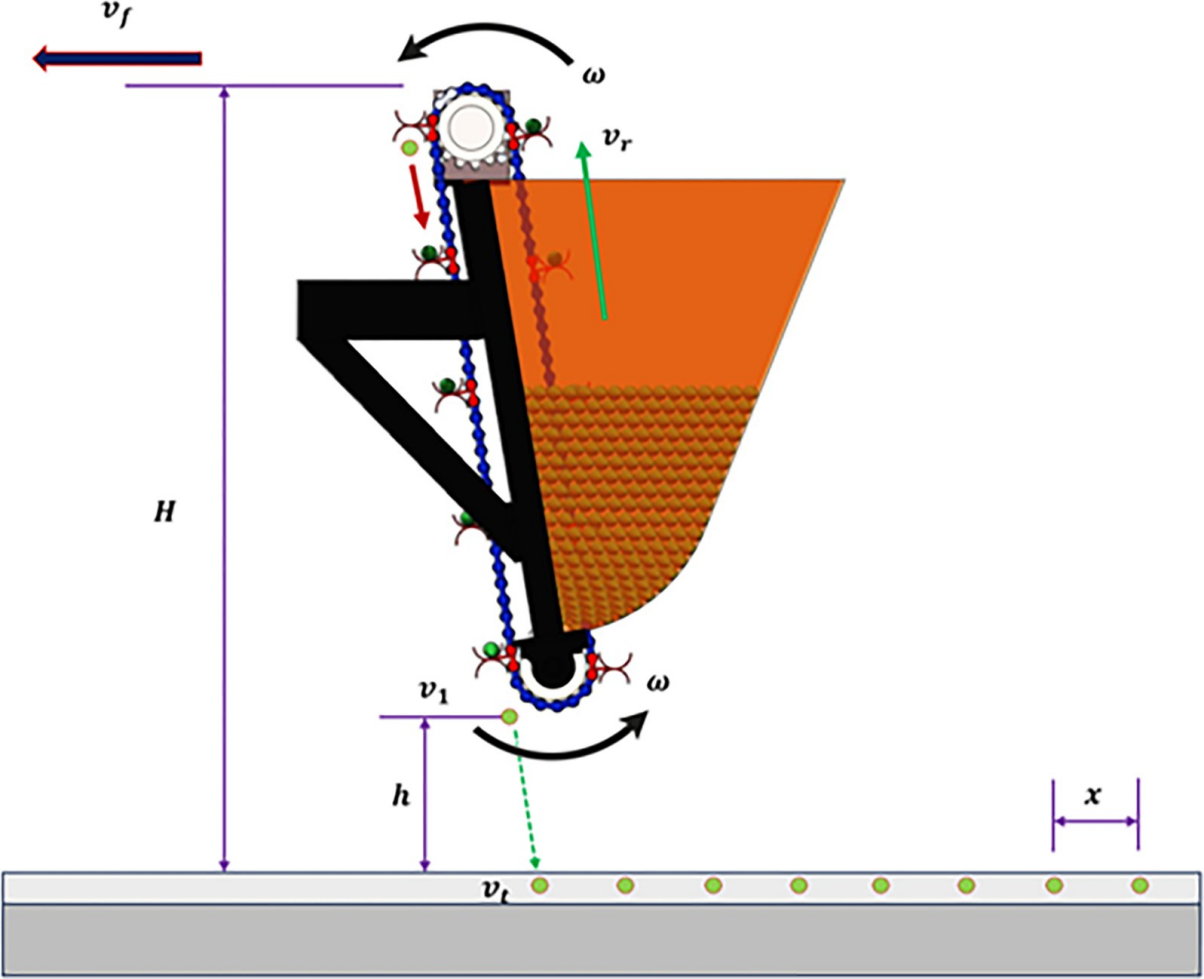
Schematic drawing of smooth transport and delivery of garlic seed during the developed EDMS. Whereas v_f_ is the travel speed, m/s; ω is the angular velocity of the stepper motor, rad/s; v_r_ is the linear speed of the feeding fork, m/s; v_1_ and v_t_ are the initial and final falling speeds of garlic seed, m/s; H is the vertical distance between the stepper motor and soil surface; cm, h is the vertical distance between the lower sprocket and soil surface; cm, and x is the seeding spacing, cm.

The implemented EDMS features an extended delivery system, which effectively reduces the drop height of the garlic seed and minimizes the frequency of bouncing collisions between the seed and the soil during high-speed precision seeding. The precision of seed placement, consistency, and alignment in both horizontal and vertical directions are enhanced. During the seeding operation, the stepper motor is responsible for driving the sprocket to rotate. Simultaneously, the feeding fork rotates the single garlic seed in a counterclockwise direction as shown in **Fig 6.** The single garlic seed is subsequently carried smoothly to the seed guide tube for sowing. The initial migration process has been executed. The garlic seed is deposited onto the seed guide chain, which rotates in time with the EDMS through the seed guide tube. It then glides effortlessly to the seed delivery point due to the combined forces of gravity and support. The garlic seed is brought to a complete stop when it is placed in the soil in a low position. The second migration operation has been executed. The velocity at which the garlic seeds descend into the seed ditch is counterbalanced by secondary distribution, resulting in efficient and seamless seed transportation and delivery, as depicted in **Fig 6**.

**Fig 7:**
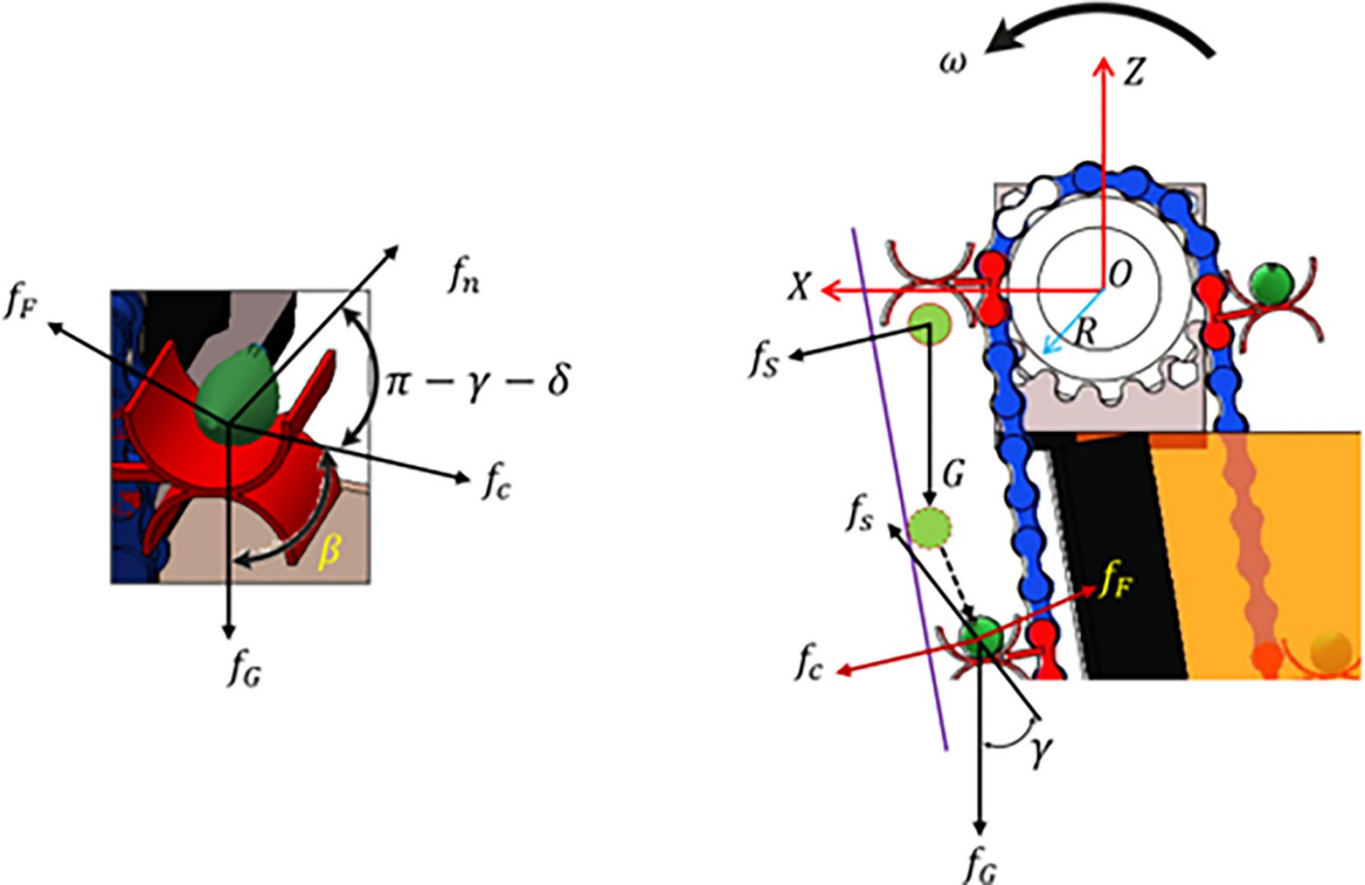
Force analysis diagram of smooth transport and delivery of garlic seed during the developed EDMS.

In order to examine the stability of the garlic seeds during the transportation and delivery process, the researchers conducted a mechanical analysis of the movement of garlic seeds. They also investigated the conditions under which the seeds and the seed guide blade maintain a relative balance [57]. By considering the revolving center of the EDMS shaft as the coordinate origin (O), a spatial Cartesian coordinate system is formed with axes X, Y, and Z, as depicted in **Fig 7**. The force exerted by a single garlic seed is examined as it rotates counterclockwise with the seed guide chain upon entering the seed guide tube. The garlic seeds are influenced by an intricate system of forces, which includes the centrifugal force (f_c_), the supporting force of the blade (f_s_), the friction force of the blade (f_F_), and the force of gravity acting on the garlic seeds (f_G_). To maintain the proper balance between the seed and the feeding fork, it is important to prevent the seed from being thrown away from the feeding fork. This ensures that the force acting on the garlic seed in the slip direction relative to the feeding fork is satisfied within the rotating plane (XOZ) of the seed guide chain.

$$f_{c}+[f_{G}\times cos\beta ]+[f_{s}\times cos(\pi -\gamma -\beta )]\leq [f_{F}\times cos\gamma ]$$ (1)

where,

$$\left\{ \begin{array}{l} f_{c}=m\times \omega^{2}\times R\\ f_{s}=m\times g\times sin\beta \\ f_{F}=\mu \times f_{s} \end{array}\right.$$ (2)

where, m is the garlic seed mass in g; β is the angle of rotation of the feeding fork in degree; γ is the inclination angle of the feeding fork in degree; μ is the coefficient of friction between the garlic seed and feeding fork, set to 0.36; R is the sprocket radius of the stepper motor in mm.

Eq 3 was generated from Eqs (1 and 2),

$$\left[m\times \omega^{2}\times R]+[f_{G}\times cos\beta ]+[m\times g\times sin\beta \times cos(\pi -\gamma -\beta )]\leq [\mu \times f_{s}\times cos\gamma \right]$$ (3)

Then, Eq (3) was simplified to Eq (4).


$$\left[m\times \omega^{2}\times R]+[m\times g\times cos\beta ]\leq [m\times g\times sin\gamma (\mu \times cos\gamma +cos(\gamma +\beta )\right]$$ (4)

In order to determine the maximum velocity of the EDMS in the critical slip condition, Eq (4) is rearranged as follows,

$$\omega \leq \sqrt{\frac{\left[g\times sin\gamma (\mu \times cos\gamma +cos(\gamma +\beta )]-[g\times cos\beta \right]}{R}}$$ (5)

When the velocity of the EDMS meets the conditions stated in Eq (5), both the garlic seed and the SMS stay in a state of minimal movement, and there is no occurrence of relative slip. Eq (5) demonstrates that the speed limit of smooth seed movement and delivery is influenced by the relative rotation angle of the feeding fork, the structural inclination angle of the feeding fork, and the radius of the stepper motor sprocket. Furthermore, this limitation is associated with the friction coefficient between the garlic seeds and the feeding fork, but it is not influenced by the bulk of the garlic seeds.

#### 2.3.3. Design of the GSMCS

**Fig 8:**
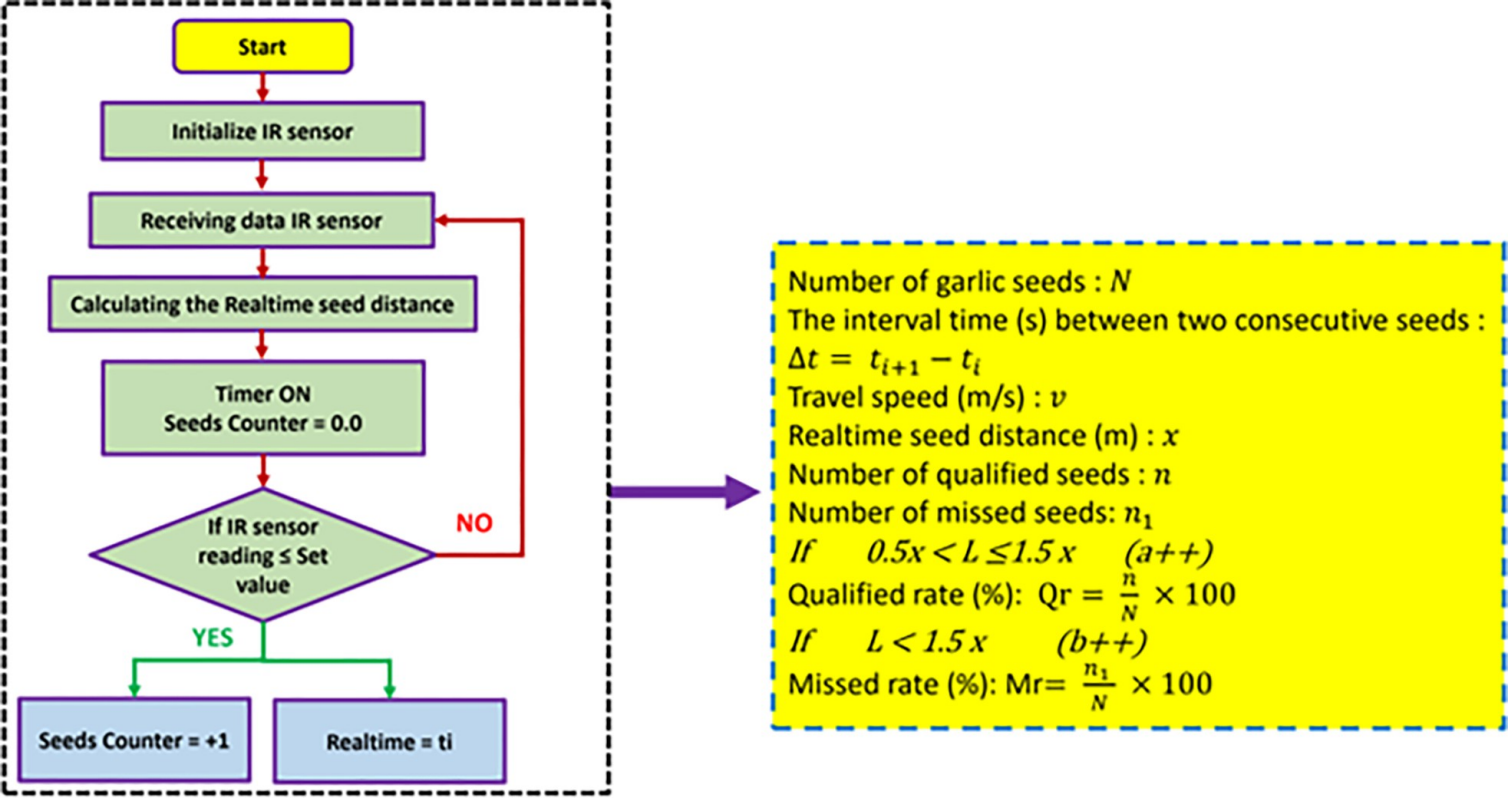
Working steps and operating algorithm of the developed GSMCS.

The GSMCS serves as an intermediary hub that connects various sensors and the terminal display software. Its primary function involves two aspects: firstly, it calculates and processes the acquired electric signals to derive the desired parameters; secondly, it enables two-way real-time communication with the terminal display software, allowing for the transmission of seeding parameters for real-time display, as well as the retrieval of parameters from the monitoring terminal. This study utilizes an Arduino board to create a controller for the CPU. The controller consists of two primary components: hardware construction and software program algorithm. The implementation of the controller’s principal function is depicted in **Fig 8**. As shown in **Fig 8**, in this study, an Arduino board was used for designing the control unit of the GSMCS that was used in the current study. Where the control unit receives the output signal from the IR sensor when the garlic seed passes through the seed tube, then the Arduino Mega board processes the signals, and then the processed signal is transferred to the laptop via the Wi-Fi module (model: Espressif Systems, Shanghai, China). The software program runs to calculate the different parameters (qualified seeds, the number of missed seeds, QR, and MR), and finally, the different calculated parameters are presented on the laptop screen.

Where the GSCMS was designed using an infrared (IR) distance sensor (model: LM393, Texas Instruments, Texas, USA) with an effective measuring distance ranging between 10–300 mm. Then the Wi-Fi module is used to transfer measured data of travel speed, the number of garlic seeds, the number of qualified seeds, the number of missed seeds, QR, and MR from the Arduino Mega board to the laptop.

### 2.4. Experiment design

The effectiveness of the developed EDMS in the current study was evaluated under laboratory conditions at the Agricultural Engineering Department, Aswan University, Egypt. The laboratory tests were conducted during April 2024. An evaluation was conducted on the EDMS to assess the accuracy of the GSMCS. A bench experiment was performed to test the seeding performance. The experimental variety chosen was the Egyptian Baladi variety of garlic seed. The average diameter is 3.73 cm, the average surface area is 85.855 cm^2^, the average quantity of seeds varies from 34 per bulb, the average bulk density is 949.5 kg/m^3^, the average repose angle is 43.28°, the average coefficient of contact is 1.015, the average coefficient of friction is 0.36, and the average crushing load is 105.3 N [58]. The performance experiment bench of the EDMS was performed by the Agricultural Engineering Department of Aswan University in Egypt. The experimental variables were chosen as the rotational speeds of the EDMS, which were 10, 20, 30, 40, 50, and 60 rpm, respectively. The experimental indices used to assess the seeding stability of the EDMS were the qualified garlic seeds, missed garlic seeds, QR, MR and coefficient of variation. In addition, the precision of the GSMCS, as well as the evaluation of the IR sensor, were conducted. The experiment was replicated at least three times, and the outcomes were averaged. Throughout the experiment, we measured the distance between neighboring garlic seeds and counted the total number of garlic seeds in order to determine each parameter. The calculating equations of the QR and MR are depicted in **Fig 8**.

The EDMS was securely installed in the mainframe during the experimental procedure. The bed belt exhibited a counter-directional movement in relation to the seed metering apparatus. A simulation was conducted to model the forward movement of the garlic planter. The fuel injection pump emitted oil onto the timing belt. The garlic seeds dropped from the sowing tube onto the garlic seedbed belt. The control unit was used to perform real-time detection and data collection in order to precisely quantify the seeding performance parameters.

### 2.5. Statistical analysis

The analysis involved the use of the commercial statistical program IBM SPSS Statistics (25) to conduct both the analysis of variance (ANOVA) and descriptive statistics. The ANOVA was specifically employed to assess the potential impact of the operating velocities of the EDMS and the GSMCS on the output results, with significance being tested at the 0.01 level.

## 3. Results and discussion

### 3.1. Calibration of the IR sensor

**Fig 9:**
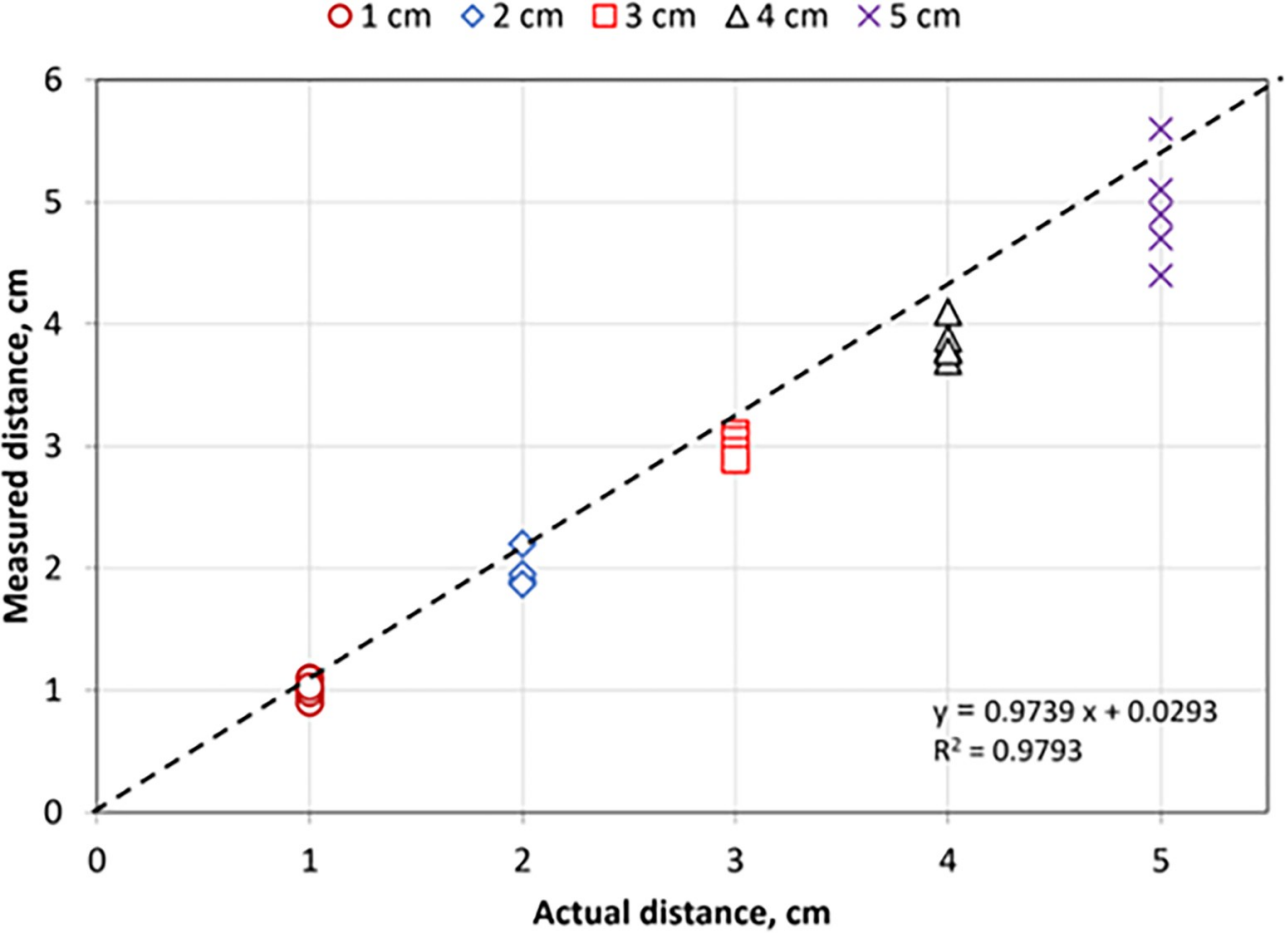
Scatter plot of the actual and measured values at each theoretical distance using IR sensor.

As described in the materials and methods section, the IR sensor was used to design an accurate GSMCS to detect garlic seed flow rate. The first step before using an electronic circuit in any application is that it must be calibrated under laboratory conditions. Where calibration is a fundamental step in ensuring the quality and trustworthiness of sensor and electronic circuit measurements. It is essential for maintaining system performance, meeting regulatory requirements, and making informed decisions based on accurate data [41,52,59,60]. The IR sensor (model: LM393, China) was calibrated under laboratory conditions at Aswan University at an air temperature of 37°C. A high-accuracy digital laser distance meter (model: Crown CT44032, USA) was used during the calibration process; the measuring range of this meter is 0.05–40 m, and the measuring accuracy is ± 2 mm. The calibration process was conducted at different distances of 1.0, 2.0, 3.0, 4.0, 5.0, and 6.0 cm. Each test was conducted at least three times. The obtained results of measured distances were plotted against actual distances (**Fig 9**). Also, **Tables 1 and 2** show the statistical analysis and ANOVA of the calibration results of the IR sensor. **Fig 9** shows a high correlation between actual and measured distances with R^2^ = 0.98 at p ≤ 0.001. Which means the measured distance is very close to the real distance. With robust linear regressions (y = 0.9739x + 0.0293) that nearly overlapped the 1:1 line, the LM393 sensor demonstrated outstanding performance at various distances.

**Table 1:** The statistical results of the calibration results of the IR sensor.

Theoretical distance, cm	Differential mean	Standard deviation	Standard error	Differential 95% confidence interval		Minimum distance	Maximum distance
				Lower limit	Upper limit		
1	1.0083	0.07935	0.03240	0.9251	1.0916	0.90	1.10
2	1.9740	0.13088	0.05853	1.8115	2.1365	1.87	2.20
3	2.9800	0.09247	0.04135	2.8652	3.0948	2.89	3.10
4	3.8520	0.15271	0.06829	3.6624	4.0416	3.70	4.10
5	4.9400	0.45056	0.20149	4.3806	5.4994	4.40	5.60

**Table 2:** ANOVA results of the calibration results of the IR sensor.

S.O.V.	Sum of Squares	df	Mean Square	F	Sig.
Between Groups	51.115**	4	12.779	258.158	≤ 0.001
Within Groups	1.039	21	0.049		
Total	52.154	25			

** highly significant at P ≤ 0.001.

**Table 1** shows the statistical analyses of the calibration results of the IR sensor at different measured distances. The illustrated data showed that the values of both standard deviation and standard error were increasing with increasing measured distance, where the maximum standard deviation and standard error were 0.45056 and 0.20149, respectively, at p-value ≤ 0.05. **Table 2** displays the analytical findings of the same statistical tests run between the IR sensor’s estimated distance measurements. At p ≤ 0.01, the statistical results showed that there is a highly significant difference between them.

Many researchers used the LM393 sensor in many fields, where in the study conducted by Hardianto et al. [61], an automatic gallon pump based on the LM393 sensor has been developed; also, an attendance system was designed and developed based on the Arduino Uno R_3_ using an LM393 sensor to detect and count students in the room [62]; additionally, Sokullu [63] developed a simple system that uses Wi-Fi signals and IR sensors to determine the number of people or animals in a predefined area; furthermore, it was used to develop an Arduino line follower using fuzzy logic control. Additionally, many types of sensors were used in designing and developing seed monitoring and counting systems [64]. On the other hand, many researchers used the ultrasonic sensor in many fields; for example, it was used to develop a remote smart monitoring system for a precision sugarcane transplanter based on IoT technology [41]; also, in the study performed by Yang et al. [43], an ultrasonic sensor integrated with a Wi-Fi module was installed in the sugarcane seed exit path for counting and monitoring the number of sugarcane seeds.

### 3.2. Evaluation the accuracy of GSMCS

**Fig 10:**
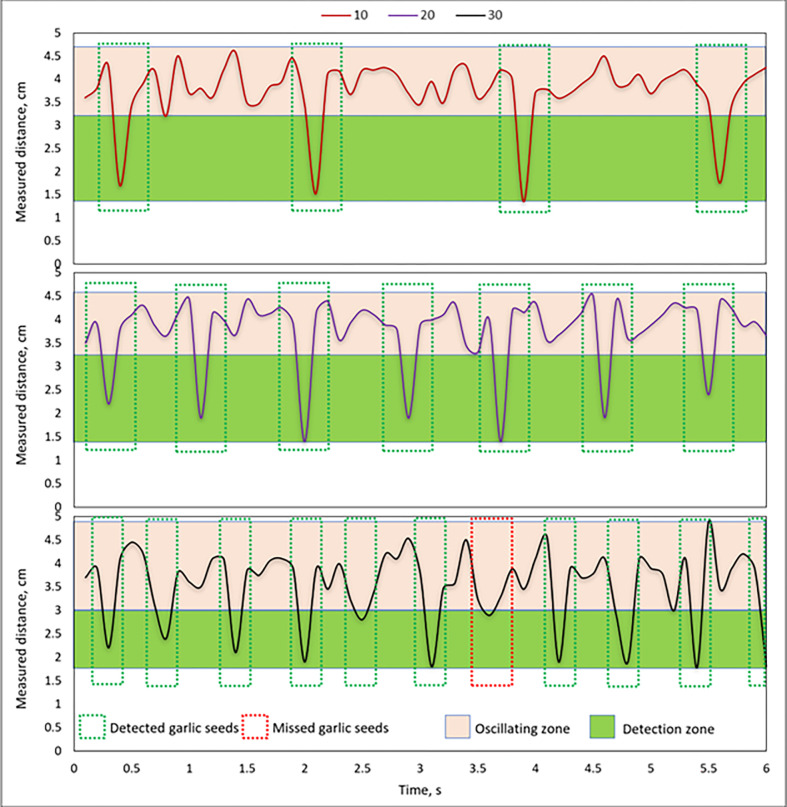
Output signals from the GSMCS under different theoretical operating velocity.

**Fig 10** shows the output signals from the GSMCS under different theoretical operating velocities of the EDMS. Where the output signals from the GSMCS were experimented with a five-rotational speed of the EDMS, these speeds were 10 rpm, 20 rpm, 30 rpm, 40 rpm, 50 rpm, and 60 rpm for 6 s, and the output signals of measured distances were observed and plotted at the y-axis and the time consumed was plotted at the x-axis (**Fig 10**). In **Fig 10**, the oscillating zone of the output signal was a red-colored area, while the green-colored area represented the useful output signals that were generated due to cutting the garlic seeds with the IR waves. Where the number of pulses in the green-colored area refers to the number of garlic seeds with respect to a specific time (seed/s). The average time interval between two garlic seeds at the operating velocity of the EDMS of 10 rpm, 20 rpm, 30 rpm, 40 rpm, 50 rpm, and 60 rpm were 1.718 s, 0.859 s, 0.573 s, 0.43 s, 0.344 s, and 0.286 s, respectively. The illustrated data in **Fig 10** presented only the output signals for the operating velocity of the EDMS of 10 rpm, 20 rpm, and 30 rpm. The results illustrated in **Fig 10** show the impact of three slower speeds of the EDMS (10 rpm, 20 rpm, and 30 rpm). Where we found that increasing the speed of the EDMS causes the pulses from the sensor unit to converge. At speeds above 30 rpm, there is significant crowding, leading to confusion and a lack of clarity in the graph. Therefore, it sufficed to show the results of the three slower speeds as an example of the shapes of the output signals from the GSMCS. Additionally, the presented data in the same figure showed that the red-colored zone increased with increased operating velocity of the EDMS, while the green-colored zone decreased. Due to the decreasing of the green-colored zone, the width of output pulses was decreased. That causes the miss-detection of garlic seeds and reduces the detection percentage (efficiency) of the GSMCS.

### 3.3. Influence of the operating velocity of the EDMS on the monitoring accuracy of the GSMCS

**Fig 11:**
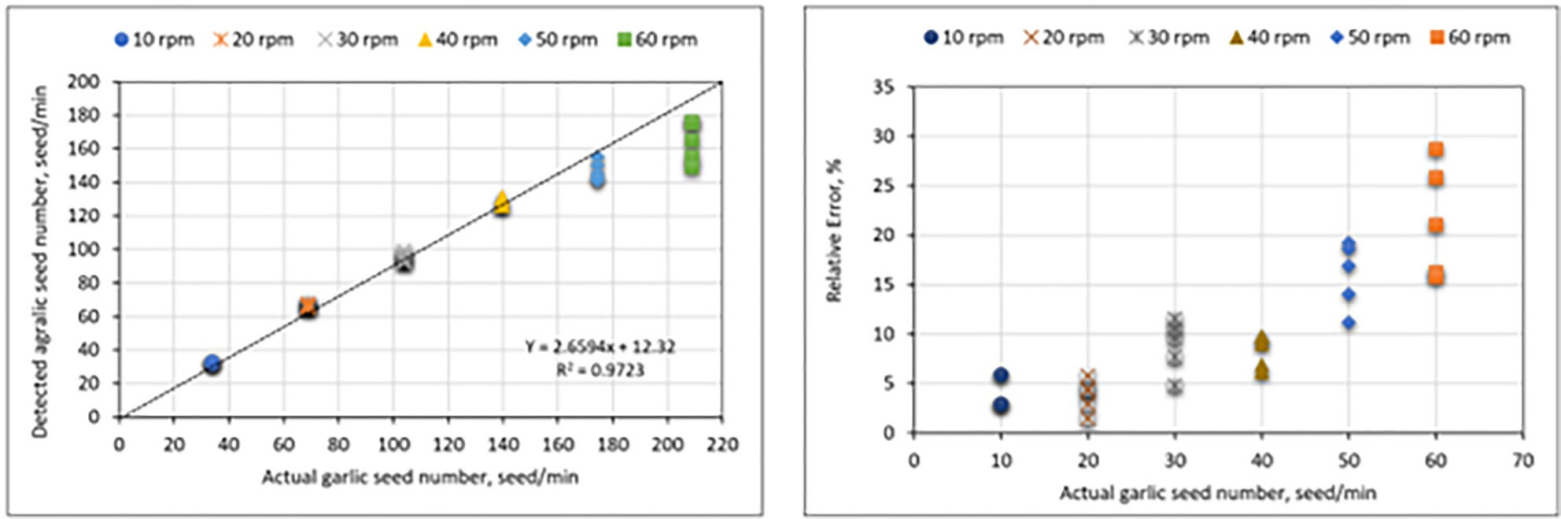
Scatter plot of the detection garlic seed numbers versus actual garlic seed numbers at different seeding flow rate.

**Fig 11A** presents the relationship between the actual and detected garlic seeds. The EDMS operated at various velocities of 10 rpm, 20 rpm, 30 rpm, 40 rpm, 50 rpm, and 60 rpm for 6 seconds, with garlic seed flow rates of 34, 69, 104, 140, 175, and 210 seeds/min. The EDMS was operated at each operating velocity, and the detected garlic seed numbers were calculated using the values acquired from three independent experiments. The detected values of garlic seed flow rate were around the actual garlic seed flow rate at the operating speeds of the EDMS of 10 rpm, 20 rpm, 30 rpm, and 40 rpm. However, increasing the operating speed of the EDMS led to an increase in the overall flow rate of the machine, but the presented data in **Fig 11A** showed a significant difference between the detected and actual flow rate of garlic seeds at operating speeds of 50 rpm and 60 rpm. On the other hand, the presented data in **Fig 11B** shows a direct relation between the relative error between the different measurements of the garlic seed flow rate at the same operating speed of the EDMS, where the relative error increases due to increasing the operating speed of the EDMS. The relative error is low, approximately 10%, when the operating speed of the EDMS is less than 40 rpm. While the relative error increased rapidly as the operating speed increased, it reached a maximum value of approximately 30% at 60 rpm.

Therefore, it is evident that increasing the operating speed of EDMS can indeed result in a decrease in detection efficiency and an increase in relative error. Numerous prior studies and researchers have ascribed this phenomenon to a multitude of factors, which may be encapsulated as follows: 1. As operating speed increases, the electronic components generate higher frequency signals, which are more susceptible to noise and interference, also, the increased noise can drown out the weak signals generated by the seeds, making detection difficult [65]. 2. Sensors used for seed detection have inherent response times. At higher speeds, the sensor might not be able to accurately capture the seed’s presence or characteristics. Additionally, the sensor’s sensitivity may decrease at higher speeds, which could hinder its ability to detect small or low-contrast seeds [66–68]. 3. Faster operating speeds generate more data, which can overwhelm the data acquisition system. Also, the processing unit might not be able to keep up with the increased data rate, leading to missed detections or inaccurate measurements [69–71]. 4. Higher speeds can cause increased mechanical vibrations in the metering device, affecting the accuracy of seed detection. Additionally, the increased stress on components due to vibrations can lead to premature wear and reduced performance [72–74]. 5. At higher speeds, the seed flow might become less uniform, making it difficult to accurately measure the seed quantity. Moreover, increased speed can cause damage to the seeds, affecting their physical properties and detection [75,76]. 6. The detection algorithms might not be optimized for high-speed operation, leading to decreased accuracy. Furthermore, software limitations can hinder the efficient processing of large amounts of data generated at high speeds [77,78]. Therefore, the development of any system must consider advanced sensor technologies, high-speed data acquisition systems, robust signal processing algorithms, and careful mechanical design to mitigate these issues.

To clarify the significance of the monitoring accuracies of the GSMCS under different seeding flow rates of 34, 69, 104, 140, 175, and 210 seeds/min, IPM SPSS Statistics version 25 software was used to analyze the single factor variance in the quantity of garlic seeds passing over the sensor with respect to time, as shown in **Tables 3 and 4**. The P values of the different operating speeds or seeding flow rates were all less than 0.0001. The results showed that the operating speeds or seeding flow rate had a highly significant influence on the evaluation parameters. The mean actual seeding flow rate was 32.6, 66.4, 94.8, 128, 146, and 164 seeds/min at operating velocities of 10 rpm, 20 rpm, 30 rpm, 40 rpm, 50 rpm, and 60 rpm, respectively. Also, the illustrated data showed that the values of both standard deviation and standard error were increasing with increasing the operating speed or seeding flow rate, where minimum and maximum values of both standard deviation and standard error were (0.54772 and 11.95826) and (0.24495 and 5.34790), respectively, at 95% confidence interval.

**Table 3:** The statistical results of the GSMCS.

Operating velocity, rpm	Differential mean of seeding flow rates	Standard deviation	Standard error	Differential 95% confidence interval		Minimum seeding flowr ates, seeds/min	Maximum seeding flow rates, seeds/min
				Lower limit	Upper limit		
10	32.6000	0.54772	0.24495	31.9199	33.2801	32.00	33.00
20	66.4000	1.14018	0.50990	64.9843	67.8157	65.00	68.00
30	94.8000	2.77489	1.24097	91.3545	98.2455	92.00	99.00
40	128.0000	2.34521	1.04881	125.0880	130.9120	126.00	131.00
50	146.6000	5.85662	2.61916	139.3280	153.8720	141.00	155.00
60	164.0000	11.95826	5.34790	149.1519	178.8481	149.00	176.00

**Table 4:** ANOVA results of the GSMCS.

S.O.V.	Sum of Squares	df	Mean Square	F	Sig.
Between Groups	62876.800**	5	12575.360	392.775	≤ 0.001
Within Groups	768.400	24	32.017		
Total	63645.200	29			

** highly significant at P ≤ 0.001.

### 3.4. Influence of the operating velocity of the EDMS on the monitoring results of the GSMCS

**Fig 12:**
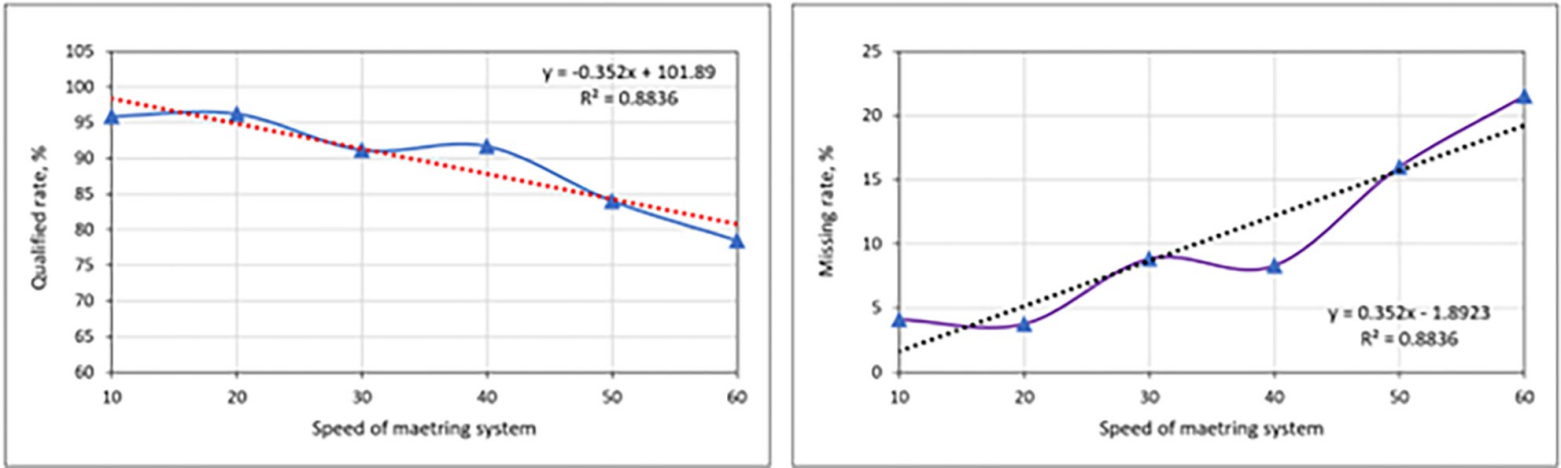
Influence of the operating velocity of the EDMS on the monitoring results of the GSMCS.

Laboratory experiments of the operating velocity are conducted to clarify the influence of the EDMS on the monitoring results of the GSMCS. The findings of the QR and MR for the garlic seeds at varied operating velocities of the EDMS are shown in **Fig 12**. The obtained data plotted revealed that the QR and operating velocity of the EDMS are inversely related. Also, the QR and MR, two evaluation parameters, follow the same law of change as the operating velocity: between 10 and 20 rpm, the results are relatively stable with a QR of more than 95%; between 30 and 40 rpm, the results are within an acceptable range; and between 50 and 60 rpm, the monitoring results fluctuate greatly. This demonstrates that the sensor monitoring accuracy is stable and high within the operating velocity range of 10–20 rpm, but unstable and low within the operating velocity range of 50–60 rpm. In addition, the QR is optimal when the EDMS operating velocities are between 10 and 20 rpm. It is evident from this that the monitoring findings are more affected by a modest change when the operating velocity is minimal. As the EDMS’s operating velocity increases, the GSMCS’s monitoring accuracy will decrease. The analysis is conducted because there is a direct correlation between the operating velocity, the rotation speed of the EDMS, and the quantity of garlic seeds released per unit time. The velocity of garlic seed movement concurrently accelerates, causing a reduction in the monitoring precision of the GSMCS. This impact is particularly notable when the EDMS operates at high velocity, adversely affecting the monitoring accuracy of the GSMCS.

Several studies have shown similar findings, including Xie et al. [75] observed a noticeable change in sensor monitoring accuracy when the seeding speed is high and the seeding spacing is small. Further investigation revealed that the rate at which seeds pass through the sensor is the main factor affecting the accuracy of sensor monitoring. As the frequency increases, the precision of sensor monitoring gradually decreases, especially after reaching 25.92 seeds per second, where the accuracy of the sensor monitoring significantly declines, showing a marked and abrupt change; in the study performed by Tang et al. [79], the results showed that as the rotational speed of the seed metering tray increased, there was a progressive rise in both the miss-seeding index and the amount of seeds being sown. Furthermore, there was a gradual decrease in the accuracy of the sensor monitoring. The precision of sensor monitoring decreased with the higher frequency and speed of sowing; in another study conducted by Li et al. [80], a roller-wheel rice SMS was developed and evaluated. The results showed that increasing the rotation speed of the SMS to 20–30 r/min improved the QR but decreased the seeding rate. However, when the rotation speed exceeded 30 r/min, there was a sharp decline in the QR and a significant increase in the reseeding rate. This was due to the rotational movement of the seeding wheel as it entered the seeding area, causing a change in the track arc, which triggered the forced seeding mechanism and generated a vibrating impact on the SMS. The increased vibration enhanced seed fluidity and facilitated the insertion of rice seeds into the hole. Future research will further explore the impact of the vibration of the seeding wheel on seeding performance. It was also observed that the rate of missing items was directly proportional to the speed of rotation, and excessive rotation speed led to inadequate time for filling the seed.

In order to determine the importance of monitoring accuracies of the GSMCS at various seeding flow rates (34, 69, 104, 140, 175, and 210 seeds/min), we utilized IPM SPSS Statistics version 25 software to analyze the variance in the quantity of garlic seeds passing over the sensor with respect to time. The detailed results of this analysis are available in **Tables 5 and 6**. All the P values for the various operating velocities or seeding flow rates were below 0.001, indicating that the impact of the operating speeds or seeding flow rate on the evaluation parameters was highly significant. The average seeding flow rates were determined to be 32.6, 66.4, 94.8, 128, 146, and 164 seeds/min at operating speeds of 10, 20, 30, 40, 50, and 60 rpm, respectively. Our data illustrated that both the standard deviation and standard error values increased as the operating speed or seeding flow rate increased. At a 95% confidence interval, the minimum and maximum values for the standard deviation were 0.54772 and 11.95826, respectively, while the standard error had minimum and maximum values of 0.24495 and 5.34790, respectively.

**Table 5:** The statistical results of the QR and MR.

Parameter	Operating velocity, rpm	Differential mean, %	Standard deviation	Standard error	Differential 95% confidence interval		Minimum, %	Maximum, %
					Lower limit	Upper limit		
**QR**	10	95.8840	1.61030	0.72015	93.8845	97.8835	94.12	97.06
	20	96.2300	1.65325	0.73936	94.1772	98.2828	94.20	98.55
	30	91.1520	2.66876	1.19350	87.8383	94.4657	88.46	95.19
	40	91.6900	1.67896	0.75085	89.6053	93.7747	90.26	93.84
	50	84.0120	3.35791	1.50170	79.8426	88.1814	80.80	88.83
	60	78.4680	5.72220	2.55904	71.3630	85.5730	71.29	84.21
**MR**	10	4.1160	1.61030	0.72015	2.1165	6.1155	2.94	5.88
	20	3.7700	1.65325	0.73936	1.7172	5.8228	1.45	5.80
	30	8.8480	2.66876	1.19350	5.5343	12.1617	4.81	11.54
	40	8.3100	1.67896	0.75085	6.2253	10.3947	6.16	9.74
	50	15.9880	3.35791	1.50170	11.8186	20.1574	11.17	19.20
	60	21.5320	5.72220	2.55904	14.4270	28.6370	15.79	28.71

**Table 6:** ANOVA results of the QR rate and MR.

Parameter	S.O.V.	Sum of Squares	df	Mean Square	F	Sig.
**QR**	Between groups	1226.825**	5	245.365	24.832	≤ 0.001
	Within groups	237.146	24	9.881		
	Total	1463.972	29			
**MR**	Between Groups	1226.825**	5	245.365	24.832	≤ 0.001
	Within Groups	237.146	24	9.881		
	Total	1463.972	29			

** highly significant at P ≤ 0.001.

In **Tables 5 and 6**, the statistical results of the QR show that p-values are less than 0.001 at each operating velocity of the EDMS, revealing that there is a highly significant difference between the detection values of the QR and MR within a 99% confidence interval. The tabulated results showed that the influence of the operating velocities or seeding flow rate on the evaluation parameters (QR and MR) was very significant. At operating velocities of 20–30 rpm, the differential mean of the QR ranged between 59.884% and 96.23%, respectively, where the highest value was observed at operating velocities of 20 rpm. While at these velocities range the standard deviation and standard error were the lowest, whereas at operating velocities of 20–30 rpm, the obtained values of both standard deviation and standard error were (1.61030 to 1.65325) and (0.72015 to 0.73936), respectively. The differential mean of the QR decreased by increasing the operating velocities where it achieved its lowest value of 78.4680% at an operating velocity of 60 rpm. At this velocity, the values of both the standard deviation and the standard error varied intensively. On the other hand, the obtained data showed that there is a direct relationship between differential mean, standard deviation, and standard error, where the values of these parameters were increased with increasing the operating velocity. The obtained results of these parameters ranging between (4.1160 to 21.5320%), (1.61030 to 5.72220), and (0.72015 to 2.55904), for differential mean, standard deviation, and standard error, at operating velocities ranged between 10 and 60 rpm, respectively. **Table 7** shows a comparison between the developed system in the current study and previous related works.

**Table 7:** Comparison between the developed system in the current study with previous related works.

Ref.	Crop type	Type of SMS	Technology	QR	MR
Wang et al. [81]	Garlic seeds	Finger clip plate	Traditional system	91.86%	2.71%
Ding et al. [82]	Garlic seeds	Finger clip plate	Traditional system	91.86%	2.71%
Guo et al. [83]	Garlic seeds	Adjustable-size seeding device	Traditional system	80%	---
Tang et al. [84]	Corn seeds	Long-Belt Finger-Clip	Electrical driven	75.75–84.70%	--
Liu et al. [85]		Flute-Roller Type	Electrical driven	94.12%	--
Liu et al. [86]	Corn seeds	Single-grain seed metering device	Electrical driven	98.75–91.30%	--
Proposed system	Garlic seeds	Chain metering system	Electrical driven (IoT)	96.23%	3.77%

## 4. Conclusion, recommendations and future work

An IoT-based electric-driven metering system (EDMS) has been combined with a smart garlic seed monitoring and counting system (GSMCS). The control system includes an Arduino Mega board, a stepper motor, a stepper motor controller, and a speed sensor. The GSMCS uses an IR sensor, and a Wi-Fi module connects the Arduino board to the laptop. This integration aims to modernize agriculture and rural areas by achieving automation, intelligence, and humanization. Combining autonomous driving with electronic control seeding has reduced labor intensity. During bench testing, the GSMCS was calibrated at different operating velocities of the EDMS to establish the conditions of output signals. This was done to estimate the relative error between the actual and measured flow rate of garlic seeds. In the laboratory tests of the GSMCS, increasing the speed of the EDMS beyond 30 rpm resulted in significant crowding, causing confusion and a lack of clarity in the graph. Additionally, increasing the operating speed of the EDMS led to an overall increase in the machine’s flow rate, but there was a significant difference between the detected and actual flow rates of garlic seeds at operating speeds of 50 rpm and 60 rpm. The relative error increased rapidly with increasing operating speed, reaching a maximum value of approximately 30% at 60 rpm, where the average QR and MR ranged between 95.884% and 96.23% and between 3.77% and 4.116%, respectively. Furthermore, increasing the operating velocity to 40 rpm or more necessitated a higher level of system stability to avoid system instability. Furthermore, increasing the EDMS’s operating velocity to 60 rpm resulted in a decrease in QR and MR to 78.4680% and 21.5320%, respectively.

**Recommendations:** In the laboratory experiment of the EDMS, when the operating velocity of the EDMS ranged from 10 to 20 rpm, the monitoring accuracy is stable and high, also, the QR is the best.

**Future works:** Using the developed EDMS and GSMCS to manufacture a smart electronic garlic seed planter is a permissible approach, and testing the manufactured machine in the field is also permissible. Also, we can assist the performance of the developed system by using artificial intelligence and machine learning.

## Supporting information

S1 File All supporting information for this paper is listed in this file. (DOCX)

## Abbreviations

SMS: seed metering systems
EDMS: electric-driven metering system
IR: infrared
QR: qualified rate
MR: missing rate
GSMCS: garlic seed monitoring and counting system
PAT: precision agriculture technology
VRA: Variable rate application
IoT: Internet of Things
PV: Photovoltaic
CPU: Central processing unit
ANOVA: analysis of variance
S.O.V.: Source of variance
SMS: Short message service
