# Supplementary material for: Development and laboratory evaluation of a novel IoT-based electric-driven metering system for high precision garlic planter
Source: PLoS One. 2025 Jan 17;20(1):e0317203. doi: 10.1371/journal.pone.0317203 (PMC11741619; doi:10.1371/journal.pone.0317203)
Supplement: S1 File — (DOCX) [file pone.0317203.s001.docx]

**FIGURE 9:** Scatter plot of the actual and measured values at each theoretical distance using IR sensor.

| **Measured** | **Actual** |
| --- | --- |
| 0.95 | 1 |
| 0.9 | 1 |
| 1.1 | 1 |
| 0.98 | 1 |
| 1.09 | 1 |
| 1.03 | 1 |
| 1.9 | 2 |
| 1.95 | 2 |
| 2.2 | 2 |
| 1.95 | 2 |
| 1.87 | 2 |
| 3.1 | 3 |
| 3.05 | 3 |
| 2.9 | 3 |
| 2.96 | 3 |
| 2.89 | 3 |
| 3.8 | 4 |
| 3.7 | 4 |
| 4.1 | 4 |
| 3.88 | 4 |
| 3.78 | 4 |
| 4.4 | 5 |
| 4.7 | 5 |
| 5.6 | 5 |
| 4.9 | 5 |
| 5.1 | 5 |

**FIGURE** **10:** Output signals from the GSMCS under different theoretical operating velocity.

| **Time, sec** | **10 rpm** | **20 rpm** | **30 rpm** |
| --- | --- | --- | --- |
| 0.1 | 3.6 | 3.5 | 3.7 |
| 0.2 | 3.8 | 3.9 | 3.9 |
| 0.3 | 4.3 | 2.2 | 2.2 |
| 0.4 | 1.7 | 3.8 | 4.1 |
| 0.5 | 3.4 | 4.1 | 4.45 |
| 0.6 | 3.9 | 4.3 | 4.2 |
| 0.7 | 4.2 | 3.87 | 3.1 |
| 0.8 | 3.2 | 3.65 | 2.4 |
| 0.9 | 4.5 | 4.12 | 3.8 |
| 1 | 3.7 | 4.43 | 3.6 |
| 1.1 | 3.8 | 1.9 | 3.5 |
| 1.2 | 3.6 | 4.12 | 4.1 |
| 1.3 | 4.2 | 3.98 | 4.08 |
| 1.4 | 4.6 | 3.67 | 2.1 |
| 1.5 | 3.5 | 4.43 | 3.85 |
| 1.6 | 3.46 | 4.1 | 3.74 |
| 1.7 | 3.84 | 4.13 | 4.06 |
| 1.8 | 3.94 | 4.25 | 4.1 |
| 1.9 | 4.45 | 3.9 | 3.89 |
| 2 | 3.48 | 1.4 | 1.9 |
| 2.1 | 1.52 | 4.15 | 3.89 |
| 2.2 | 4.1 | 4.39 | 3.45 |
| 2.3 | 4.18 | 3.56 | 3.99 |
| 2.4 | 3.67 | 3.94 | 3.2 |
| 2.5 | 4.19 | 4.2 | 2.8 |
| 2.6 | 4.2 | 4.1 | 3.4 |
| 2.7 | 4.25 | 3.88 | 4.2 |
| 2.8 | 4.1 | 3.78 | 4.1 |
| 2.9 | 3.7 | 1.9 | 4.53 |
| 3 | 3.45 | 3.88 | 3.8 |
| 3.1 | 3.95 | 3.99 | 1.8 |
| 3.2 | 3.48 | 4.1 | 3.46 |
| 3.3 | 4.2 | 4.35 | 3.57 |
| 3.4 | 4.3 | 3.44 | 4.5 |
| 3.5 | 3.59 | 3.3 | 3.24 |
| 3.6 | 3.78 | 3.99 | 2.89 |
| 3.7 | 4.2 | 1.4 | 3.3 |
| 3.8 | 4.01 | 4.2 | 3.88 |
| 3.9 | 1.35 | 4.15 | 3.45 |
| 4 | 3.69 | 4.36 | 4.1 |
| 4.1 | 3.78 | 3.55 | 4.56 |
| 4.2 | 3.59 | 3.67 | 1.9 |
| 4.3 | 3.69 | 3.89 | 3.89 |
| 4.4 | 3.89 | 4.15 | 3.69 |
| 4.5 | 4.1 | 4.5 | 3.78 |
| 4.6 | 4.5 | 1.91 | 4.1 |
| 4.7 | 3.89 | 4.4 | 2.89 |
| 4.8 | 3.87 | 3.59 | 1.89 |
| 4.9 | 4.1 | 3.69 | 4.1 |
| 5 | 3.69 | 3.88 | 3.89 |
| 5.1 | 3.96 | 4.1 | 3.78 |
| 5.2 | 4.1 | 4.35 | 2.99 |
| 5.3 | 4.2 | 4.25 | 4.1 |
| 5.4 | 3.9 | 4.2 | 1.78 |
| 5.5 | 3.5 | 2.4 | 4.89 |
| 5.6 | 1.75 | 4.4 | 3.45 |
| 5.7 | 3.4 | 4.23 | 3.89 |
| 5.8 | 3.9 | 3.86 | 4.2 |
| 5.9 | 4.1 | 3.95 | 3.88 |
| 6 | 4.25 | 3.66 | 1.8 |

**Figure 11.** Scatter plot of the detection garlic seed numbers versus actual garlic seed numbers at different seeding flowrate.

| **Actual garlic seed number** | **Detected garlic seed number** | **Operating speed, rpm** | **Relative error, %** |
| --- | --- | --- | --- |
| 33 | 34 | 10 | 2.941176 |
| 33 | 34 | 10 | 2.941176 |
| 32 | 34 | 10 | 5.882353 |
| 32 | 34 | 10 | 5.882353 |
| 33 | 34 | 10 | 2.941176 |
| 66 | 69 | 20 | 4.347826 |
| 67 | 69 | 20 | 2.898551 |
| 68 | 69 | 20 | 1.449275 |
| 65 | 69 | 20 | 5.797101 |
| 66 | 69 | 20 | 4.347826 |
| 99 | 104 | 30 | 4.807692 |
| 94 | 104 | 30 | 9.615385 |
| 96 | 104 | 30 | 7.692308 |
| 93 | 104 | 30 | 10.57692 |
| 92 | 104 | 30 | 11.53846 |
| 130 | 139.6 | 40 | 6.876791 |
| 127 | 139.6 | 40 | 9.025788 |
| 131 | 139.6 | 40 | 6.160458 |
| 126 | 139.6 | 40 | 9.74212 |
| 126 | 139.6 | 40 | 9.74212 |
| 155 | 174.5 | 50 | 11.17479 |
| 150 | 174.5 | 50 | 14.04011 |
| 145 | 174.5 | 50 | 16.90544 |
| 141 | 174.5 | 50 | 19.19771 |
| 142 | 174.5 | 50 | 18.62464 |
| 175 | 209 | 60 | 16.26794 |
| 176 | 209 | 60 | 15.78947 |
| 155 | 209 | 60 | 25.83732 |
| 149 | 209 | 60 | 28.70813 |
| 165 | 209 | 60 | 21.05263 |

**Figure 12.** Influence of the operating velocity of the EDMS on the monitoring results of the GSMCS.

| **Operating speed, rpm** | **Qualified rate, %** | **Missed rate, %** |
| --- | --- | --- |
| 10 | 97.06 | 2.94 |
| 10 | 97.06 | 2.94 |
| 10 | 94.12 | 5.88 |
| 10 | 94.12 | 5.88 |
| 10 | 97.06 | 2.94 |
| 20 | 95.65 | 4.35 |
| 20 | 97.10 | 2.90 |
| 20 | 98.55 | 1.45 |
| 20 | 94.20 | 5.80 |
| 20 | 95.65 | 4.35 |
| 30 | 95.19 | 4.81 |
| 30 | 90.38 | 9.62 |
| 30 | 92.31 | 7.69 |
| 30 | 89.42 | 10.58 |
| 30 | 88.46 | 11.54 |
| 40 | 93.12 | 6.88 |
| 40 | 90.97 | 9.03 |
| 40 | 93.84 | 6.16 |
| 40 | 90.26 | 9.74 |
| 40 | 90.26 | 9.74 |
| 50 | 88.83 | 11.17 |
| 50 | 85.96 | 14.04 |
| 50 | 83.09 | 16.91 |
| 50 | 80.80 | 19.20 |
| 50 | 81.38 | 18.62 |
| 60 | 83.73 | 16.27 |
| 60 | 84.21 | 15.79 |
| 60 | 74.16 | 25.84 |
| 60 | 71.29 | 28.71 |
| 60 | 78.95 | 21.05 |
